# An Inclusive Civil Society Dialogue for Successful Implementation of the EU HTA Regulation: Call to Action to Ensure Appropriate Involvement of Stakeholders and Collaborators

**DOI:** 10.3390/jmahp12010004

**Published:** 2024-03-14

**Authors:** Thomas Desmet, Elaine Julian, Walter Van Dyck, Isabelle Huys, Steven Simoens, Rosa Giuliani, Mondher Toumi, Christian Dierks, Juliana Dierks, Antonella Cardone, Francois Houÿez, Mira Pavlovic, Michael Berntgen, Peter Mol, Anja Schiel, Wim Goettsch, Fabrizio Gianfrate, Stefano Capri, James Ryan, Pierre Ducournau, Oriol Solà-Morales, Jörg Ruof

**Affiliations:** 1 Healthcare Management Centre, Vlerick Business School, 1210 Brussels, Belgium; 2 Department of Pharmaceutical and Pharmacological Sciences, KU Leuven, 3000 Leuven, Belgium; 3 Secretariat of the European Access Academy (EAA), 4059 Basel, Switzerland; 4 Guy’s and St Thomas’ NHS Foundation Trust, London SE1 7EH, UK; 5 Public Health Department, Faculty of Medicine, Aix-Marseille University, 13002 Marseille, France; 6 Dierks+ Company, 10115 Berlin, Germany; 7 Cancer Patients Europe, 1000 Brussels, Belgium; 8 European Organisation for Rare Diseases (EURORDIS), 75014 Paris, France; 9 Medicines Development and Training (MDT) Services, 75020 Paris, France; 10 European Medicines Agency (EMA), 1083 HS Amsterdam, The Netherlands; 11 Department of Clinical Pharmacy and Pharmacology, University Medical Center Groningen (UMCG), University of Groningen, 9713 GZ Groningen, The Netherlands; 12 Norwegian Medicines Agency (NOMA), 0950 Oslo, Norway; 13 Utrecht Centre for Pharmaceutical Policy, Division of Pharmacoepidemiology and Clinical Pharmacology, University of Utrecht, 3584 CS Utrecht, The Netherlands; 14 National Health Care Institute, 1110 AH Diemen, The Netherlands; 15 Department of Economics and Management, University of Ferrara, 44121 Ferrara, Italy; 16 School of Economics and Management, Cattaneo-LIUC University, 21053 Castallanza, Italy; 17 Astra Zeneca, Cambridge CB2 8PA, UK; 18 Abbvie AG, 6330 Cham, Switzerland; 19 HiTT Foundation, International University of Catalonia-UIC, 08015 Barcelona, Spain; 20 Medical School of Hanover, 30625 Hanover, Germany

**Keywords:** EUHTA, health policy, health technology assessment, stakeholder involvement

## Abstract

Objectives: Stakeholder involvement has long been considered a success factor for a joint European health technology assessment (HTA) process, and its relevance is now anchored in the EU HTA Regulation’s (EU HTAR) legislative wording. Therefore, we aimed to explore the roles, challenges, and most important activities to increase the level of involvement per stakeholder group. Methods: At the 2022 Fall Convention of the European Access Academy (EAA), working groups addressed the involvement of patients, clinicians, regulators, health technology developers (HTD), and national HTA bodies and payers within the EU HTA process. Each working group revisited the pre-convention survey results, determined key role characteristics for each stakeholder, and agreed on the most important activities to fulfill the role profile. Finally, the activities suggested per group were prioritized by plenary group. Results: The prioritized actions for patients included training and capacity building, the establishment of a patient involvement committee, and the establishment of a patient unit at the EC secretariat. For clinicians, it included alignment on evidence assessment from a clinical vs. HTA point of view, capacity building, and standardization of processes. The most important actions for regulators are to develop joint regulatory-HTA guidance documents, align processes and interfaces under the regulation, and share discussions on post-licensing evidence generation. HTDs prioritized scientific advice capacity and the review of the scoping process, and further development of the scope of the assessment report fact checks. The top three actions for national HTA bodies and payers included clarification on the early HTD dialogue process, political support and commitment, and clarification on financial support. Conclusions: Addressing the activities identified as the most important for stakeholders/collaborators in the EU HTA process (e.g., in the implementation of the EU HTA Stakeholder Network and of the guidance documents developed by the EUnetHTA 21 consortium) will be key to starting an “*inclusive civil society dialogue*”, as suggested by the European Commission’s Pharmaceutical Strategy.

## 1. Introduction

New health technologies undergo health technology assessment (HTA), a systematic and multidisciplinary evaluation of their properties covering both their direct and indirect consequences. HTA summarizes information about medical, economic, social, and ethical issues related to the use of health technology. Its aim is to determine the value of health technologies or interventions to inform guidance on how they can be used in health systems [1,2]. Due to differences in healthcare systems and willingness to invest in healthcare, differences in assessment methodologies and appraisal practices, and variations in economic constraints, discrepancies in HTA recommendations exist and may produce disparities in terms of patient access to medicines. EU-wide harmonization is thought to support alignment regarding access and reimbursement timing across Europe [3,4].

In December 2021, the European Regulation on Health Technology Assessment (EU HTAR), was adopted by the Council and the European Parliament and became effective as of January 2022 [5]. The regulation aims to harmonize methodological standards in HTA and foster collaboration among European member states’ HTA bodies at the EU level in the context of joint clinical assessments (JCA) and joint scientific consultations (JSC) [6].

In the previous work of the original European Network for HTA (EUnetHTA) project, Work Package 6 focused on stakeholder involvement and considered it necessary to ensure transparency of interests and processes, legitimacy, and wide utilization of EUnetHTA and its products [7,8]. Now, more than ten years later, the importance of comprehensive stakeholder/collaborator involvement is confirmed as a key success factor for the evolving EU HTA process by respective wording being anchored in the EU HTAR [6]. The need to establish a “*stakeholder network*” that “*shall support the work of the Coordination Group and its subgroups upon request*” is mentioned in Article 29 of the EU HTAR. Recital 44 further specifies that “*the Coordination Group should engage and consult widely with stakeholder organizations with an interest in Union cooperation on HTA, including patient organizations, healthcare professional organizations, clinical and learned societies, health technology developer associations, consumer organizations and other relevant non-governmental organizations in the field of health*” [6].

In 2021 the European Access Academy (EAA) was founded as a multi-stakeholder initiative aiming to facilitate and further support the implementation of the EU HTAR. The EAA was designed as a scientifically anchored initiative identifying and discussing the “hot topics” throughout the preparation and implementation of the regulation and proposing actionable solutions [9,10,11,12]. As stakeholder involvement had been identified as one of the critical success factors [8,10], the 2022 Fall Convention of the EAA in Brussels focused on this topic. Based on the wording of the EU HTAR, the direct impact of EU HTA procedures, and the relevance of EU HTAR outcomes, four key stakeholder groups were identified: (i) patients and patients’ representatives; (ii) clinicians, healthcare practitioners, and medical societies (clinicians’ representatives); (iii) regulators; and (iv) industry associations and health technology developers (HTD, industry representatives). National HTA bodies and payers are considered a separate group, as they are not stakeholders listed within the regulation but are considered stakeholders due to their roles in the access process, conducting the HTA assessment, and, depending on the national system, as decision-makers regarding appraisal and/or pricing and reimbursement [13]. While national HTA bodies typically assess the (comparative) effectiveness and, in some member states, also the cost-effectiveness and budget impact of health technologies (medical treatments, devices, healthcare interventions) to inform healthcare decision-making, payers can include health insurance companies or government health agencies that provide healthcare services to the general public and might, in certain contexts, also be decision-makers, pertaining to pricing and reimbursement of health technologies. Despite the separate remits of these two roles, in many cases, there is significant overlap or at least very close interconnection; hence, these two were grouped together for the purpose of this work.

While regulators are not covered in recital 44 of the EU HTAR and would be considered collaborators rather than stakeholders, they were included in subsequent analyses due to their critical role in the European market access process [6,13]. The EAA Fall Convention was preceded by the development and conduct of a multi-stakeholder questionnaire. The questionnaire results revealed an important difference between the self-perceived involvement of each key stakeholder group within the current HTA process (perceived to be very low) and respective external perceptions of the stakeholders’ involvement by HTA bodies, payers, and health policymakers (rated rather high). Respective results are reported elsewhere [13] and accumulated in a RACI chart, reflecting the level of “Responsibility”, “Accountability”, “Consultation”, and “Information” for each of the stakeholders/collaborators in the centralized procedure, as per the current EU HTAR [6,13].

Based on the insights from the pre-convention survey [13], this study aims to revisit the responses received with respect to the suggested roles and challenges for each stakeholder/collaborator group within the EU HTA process (both in the preparatory and implementation phase) and to identify the most important activities per group to increase their respective level of involvement.

## 2. Methods

The EAA aims to support the implementation of the EU HTAR and the development of a joint European value framework for the assessment of innovative health technologies by facilitating multi-stakeholder discussions. To this end, twice annually, conventions are held in an academic setting to identify and discuss challenges and to develop approaches and working agendas among the relevant stakeholders and collaborators for EU HTA to address these challenges.

### 2.1. The EAA Convention Format: Public Session and Stakeholder-Centered Working Groups

The setup of the EAA conventions includes public sessions that are widely shared via web streaming and promoted on social media. The 2022 Fall Convention took place at Vlerick Business School in Brussels, and the public session was joined by ~80 remote and ~50 onsite participants. The second part of the convention includes parallel working sessions by invite, designed as hybrid meetings, allowing both onsite and remote participation. These are followed by a concluding plenary session setting the course of action for the future.

In preparation for the discussions, various working groups were identified, and leadership teams were assigned to each working group. A key insight derived from the pre-convention survey [13] was the relevance of including academic stakeholders in the evolving EU HTA process. However, as the primary role of the EAA and the involved academic participants is rather to facilitate the “inclusive civil society dialogue”, it was decided prior to the convention not to include academic stakeholders as a separate working group, bringing the total to five dedicated working groups. The working group leadership teams, comprising two (co)-leads and a *notetaker*, included for patients: AC, FH, and *MP*; for clinicians: RG, MT, and *CD*; for regulators: MB, PM, and *JD*; for HTDs: SC, JR (James Ryan), and *PD*; and for national HTA bodies and payers: AS, WG, and *FG*. These individuals played an essential role in guiding and documenting the progress of their respective working groups.

In separate pre-convention virtual meetings, work assignments were agreed upon between the EAA secretariat and the respective working group leads. Assignment of stakeholders/collaborators to one of the five working groups was done prior to the convention to achieve a balanced distribution of various stakeholders/collaborators in each working group, consisting of 14–15 participants. Criteria for participant assignments to each of the workshops included (i) personal and professional background, (ii) including national diversity in each group, (iii) stakeholder diversity within each of the working groups, and (iv) participation mode (i.e., remote vs. onsite).

### 2.2. Procedural Approach of the Working Groups

During the working session, each of the five working groups revisited in parallel the findings of the pre-convention survey [13] and, based on these findings, identified the most important action points for each of the four key stakeholder groups: patients, clinicians, regulators, and HTDs, and, separately, for national HTA bodies and payers.

To identify and prioritize the most important activities per group, three steps were applied. First, each working group revisited the outcomes of the pre-convention survey and determined the key characteristics of each of the identified stakeholders’/collaborators’ roles. For this purpose, a content guidance document for each break-out group reflecting the relevant survey results (quantitative charts and free-text responses received prior to the convention) as input for the discussions was developed by the EAA secretariat (EJ and JR). Second, the working groups identified the most important actions to fulfill the role profile and address the related challenges. We considered the definition of “role” as the position or purpose that a stakeholder has in the EU HTA process [14].

The EAA Convention working group sessions were scheduled to offer sufficient time for discussion, allowing participants to share lessons learned, identify solutions, and crystallize priorities to move forward. Between two 90-minute sessions, a short working lunch was scheduled allowing for a *status quo* report of all working group leaders and confirmation of progress and realignment on session goals. 

### 2.3. Plenary Session and Ranking

After the working group sessions, the findings of each of the five groups were reported back to the EAA plenary group. Following presentations by each of the working groups’ leads, their suggested most important activities were discussed and formally ranked. For this, all stakeholders present in the room and online were asked to rate the presented activities by importance (prioritization). 

During the plenary session, key discussion points were reflected within the group, including the structure of the proposed activities and the wording of the respective action points, as well as feasibility and implementation challenges. 

### 2.4. Data Handling and Analysis

All rankings were conducted on an ordinal Likert response scale from 1 (low priority) to 7 (high priority). Pre-generated QR codes were shared to allow for simultaneous IT-based ranking using online forms generated with Microsoft 365 Office Online. The respective QR codes, as well as HTML links, were projected in the room and online and provided as hard copies. All ranking responses received were pseudonymized prior to any analysis in Excel. Aggregated descriptive data were simultaneously visible to the audience, allowing for an informed discussion within the concluding plenary session. Data were stored in a password-protected separate file. Finally, pre-defined data analysis was conducted independently by two of the authors (TD, EJ).

## 3. Results

### 3.1. Stakeholder-Centered Working Groups at the EAA Convention

**Figure 1:**
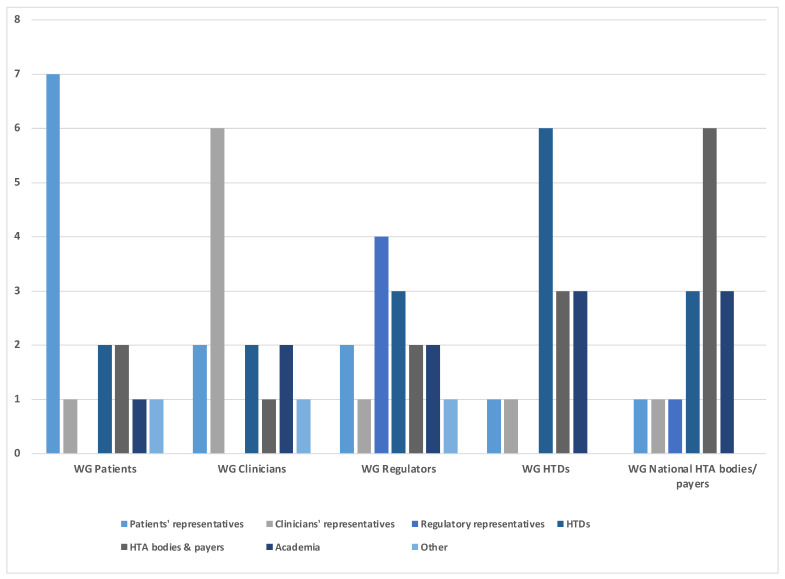
Number and background of experts participating in each of the working group sessions (WG).

The working groups at the EAA convention consisted of a total of 72 participants, with 14 participants in each of the patient, clinician, and HTD working groups, and 15 participants in each of the regulatory and HTA body working groups. Participation in each working group was mixed with a total of 13 patients, 10 clinicians, 5 regulatory bodies, 16 HTDs, 14 HTA bodies and payer representatives, 11 academics, and 3 other participants (including policymakers and subject matter experts, e.g., with previous affiliations with an institution of the stakeholder groups listed above) distributed equally across the groups. Details on the constitution of each working group are provided in Figure 1.

### 3.2. Characteristics of Role Profiles and Related Challenges

Key features of the role profile for each stakeholder and collaborator are summarized in Table 1.

- ***Patients and patient representatives***, as the end users of the health technology that is being assessed, should be considered equal-level stakeholders to be involved throughout the whole HTA process and advising on the medical and symptomatic context. Specific challenges identified for patients to fulfill this role include lack of resources/unreadiness, funding, management of conflicts of interest, educational needs, and inconsistent recognition of the value of patient input.
- ***Clinicians and medical societies*** are key advisers for incorporating clinical expertise in the HTA process. This not only includes context of the disease and medical and/or scientific background, the actual bedside experience and expertise on treatment algorithms and standard of care, but also research and methodological expertise. To fulfill their role, capacity would need to be built up, educational efforts with a focus on methodological expertise are required, and conflicts of interest should be handled transparently.
- ***Regulators*** are collaborators rather than stakeholders in the evolving EU HTA process, having different although related remits than HTA bodies. Key challenges experienced in the regulatory/HTA collaboration include capacity constraints to conduct parallel scientific consultations, confidentiality arrangements (e.g., in the context of providing information to support HTA processes), and expert involvement across regulatory and HTA reviews in light of different conflict-of-interest rules.
- ***HTDs*** contribute the evidence package for the HTA assessment. A concern was raised pertaining to available slots for the JSC being scarce and HTDs appearing to be informed rather than consulted during the currently proposed JCA procedure. Further, the timelines within the process, as currently defined, are very short. Not allowing HTDs to provide key content feedback at the European level may hinder the opportunity to reduce duplicate work at the national level. Together, the long-term goal of simplifying and harmonizing the HTA process might be missed, especially when a multiplicity of PICO data requirements may create additional complexity.

### 3.3. Most Important Actions per Stakeholder/Collaborator Group

The most important actions proposed for each of the stakeholder and collaborator groups are presented in Table 2. The identified suggestions were related to capacity building, process optimization, methodological requirements, and training/educational needs as main overarching topics.

- ***Capacity***-related actions and clarification on financial support were raised by patients’, clinicians’, regulatory, and national HTA bodies’ representatives. Furthermore, patients’ representatives asked for a structural base of involvement (the patient unit at the European Commission (EC) with the establishment of a patient involvement committee), and national HTA bodies’ representatives asked for clarity on political support and commitment.
- ***Process***-related actions were raised by clinicians’ representatives (standardization of EU HTA processes), regulatory representatives (alignment of regulatory and HTA processes and interfaces; development of joint guidance documents between regulators and HTA), HTDs (review of the PICO scoping process; further development of the assessment report fact-checking process; availability of scientific advice at scale), and national HTA bodies’ representatives (clarification on the early dialogue process; clarification on designing and establishing the interface of national vs. EU-level processes).
- ***Methodological*** issues requiring action were raised by clinicians (alignment on evidence assessment), regulatory representatives (shared discussions on post-launch evidence generation (PLEG)), and HTD representatives (elements of PICO, including data requirements and harmonization).
- ***Training and educational*** actions were highlighted by patients’, clinicians’, and national HTA bodies’ representatives.

### 3.4. Prioritization of Actions

**Figure 2:**
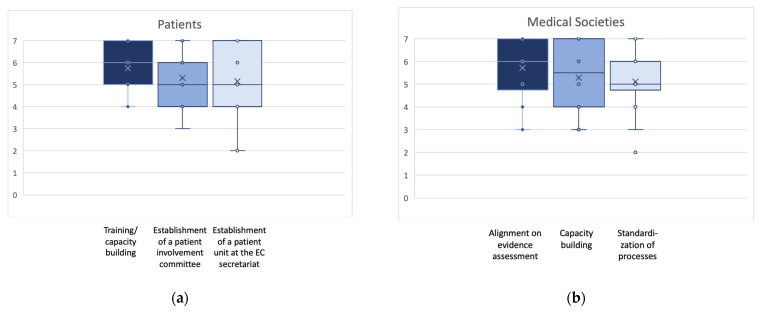

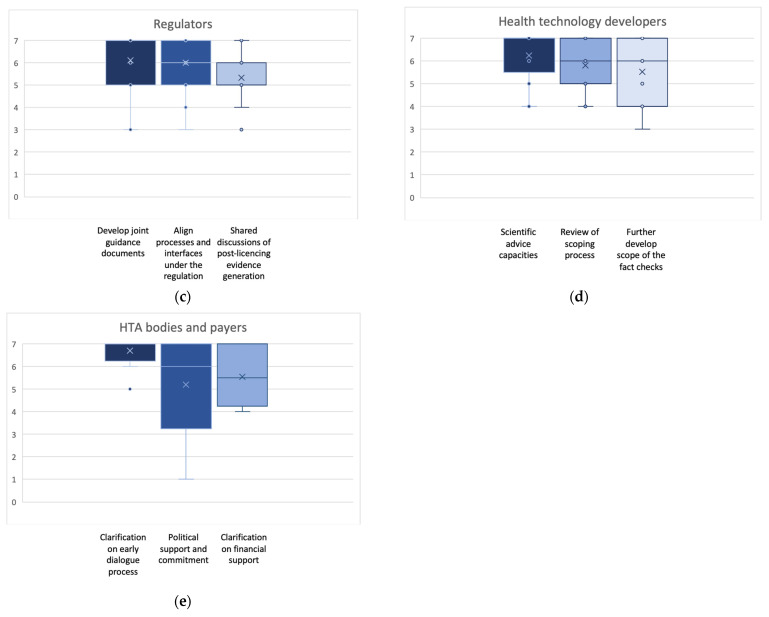
(**a**–**e**): Box plots for the ranking of the three highest ranked activities suggested to approach the identified challenges for each stakeholder/collaborator group. Indicated are mean (x); median (bar in colored area); interquartile range (colored area), any individual ranks that were chosen (dots); and min/all rankings were conducted on an ordinal Likert Response Scale scoring from 1 (low priority) to 7 (high priority).

The EAA procedural guidance for the break-out groups requested the identification of the three most important actions by each group. However, additional actions were allowed if working groups identified further actions considered of crucial importance. The final number of identified actions was three for patients’, clinicians’, and HTD representatives each, four for regulators, and five for national HTA bodies’ and payer representatives. Figure 2a–e display the outcomes of the final plenary prioritization of the most relevant three key actions per stakeholder and collaborator group. The additional actions not shown in Figure 2 are sufficient capacity as a relevant action point for regulators (scale 1–7: mean 4.8; SD 1.3; median 5.0; min 3, max 7), and for national HTA bodies and payers, training and education (scale 1–7: mean 5.3; SD 1.2; median 5.0; min 3, max 7) and participation in designing the EU HTA process (scale 1–7: mean 5.3; SD 1.2; median 5.0; min 3, max 7).

## 4. Discussion

In the final chapter of the European Commission’s Pharmaceutical Strategy for Europe [15], it is suggested that “*an **inclusive civil society dialogue** building on existing structures will be used to facilitate the interaction with stakeholders*”. While the EU HTAR is only one component of the overall EC Pharmaceutical Strategy, the evolving European methodological deliverables, JSC and JCA procedures, and related transversal activities will shape Europe’s market access for innovative medicines and medical devices in the mid to long term. Therefore, the conduct of “*an inclusive civil society dialogue*” covering the remits of the EU HTAR seems of particular importance. Deliberative processes including stakeholder participation for informed HTA decision-making vary significantly within European countries depending on their respective national processes [16,17,18,19,20]. The multiplicity of existing and envisioned levels of participative practices poses further challenges to achieving such an “*inclusive civil society dialogue*” within the EU HTA procedure.

As a first step to facilitate this ambition, we issue a call to action to promote the adequate involvement of each type of stakeholder/collaborator, as discussed below:

***Patients*** and the ultimate goal to improve patients’ lives constitute the “raison d’être” for any pharmaceutical strategy. Therefore, the call to action identified during the EAA working session aims to enable the appropriate involvement of patients and patient representatives adding patient relevance to contextualize HTA. However, structural and educational limitations need to be addressed to ensure a strong voice of patients in the developing European civil society dialogue. Of note, in early 2022, the EMA published an update to its Engagement Framework covering the interaction between the EMA and patients [21]. Therefore, we call for a similar framework, including structure and sufficient capacities, to be considered for balanced patient involvement in the EU HTA process.

Patient involvement in decision-making was included as an element of the suggested role profile defined in the respective working group. Careful consideration of all ramifications of such a request from a patient, political, and legal perspective is required, as previously discussed on a national level in the context of the German AMNOG system [22,23,24]. Also, several European countries are considering approaches to involve patients in HTA decision-making (e.g., Belgium) or have implemented approaches to do so (e.g., NICE in England and Wales, SMC in Scotland). Hence, we propose that best practices are identified and inform how patients are involved in the implementation of the EU HTAR.

**Clinicians’, healthcare practitioners’, and medical societies’** input will be key to ensure an adequate and successful implementation of the EU HTAR. Different from HTA bodies, which primarily rely on clinical data provided in the form of a dossier by the HTD to assess the clinical and economic value of health technologies to inform decision-making, clinicians’ input provides clinical context and relies on all three pillars of evidence-based medicine (clinical data; clinicians’ experience; patient status and preferences) [25]. The paradigm shift towards targeted medicine leads to specific challenges, which, for example, also affect many oncological and advanced therapeutic medicinal products (ATMPs). These will be the initial focus of the EU assessments from 2025, and adequate evidence assessments (e.g., as represented in ESMO’s Magnitude of Clinical Benefit Scale) are needed [26,27,28,29]. It has been argued that [30] medical societies’ representatives are the key connecting elements across the three pillars of market access processes that rely on clinical guidelines reflecting evidence-based medicine, necessary for benefit/risk (i.e., regulatory) and HTA assessment. Action is required to ensure sufficient capacity and standardization of processes (e.g., the development and maintenance of up-to-date—living—guidelines and their harmonization at the European level to shape the EU HTA PICO scheme) to allow comprehensive medical societies’ input in developing EU HTA PICO requirements, and more broadly support a civil society dialogue. Also, clinicians should receive adequate funding by a transparent system to ensure sufficient capacity for the workload.

As the clinical parts of the assessments by **regulators** and HTA bodies largely rely on the same clinical data, alignment of processes, development of joint advice and methodology and disease-specific guidance documents, and shared discussions on evidence generation issues are called for. These key elements were already outlined in the 2022 joint work plan between the EMA and EUnetHTA 21 [31]. However, after EUnetHTA 21 activities terminated in 2023, the EU HTA Coordination Group took over responsibility for driving these tasks.

Another important element of collaboration between regulatory and HTA bodies relates to experience sharing. As the EC’s declared intention is “*to avoid multiple assessments of a product in different Member States and improve the functioning of the Single Market for health technologies*” [6], we call for utilizing the important opportunity of learning from the experience gained by national regulatory bodies when developing a harmonized European view on clinical evidence in the context of the EMA’s establishment [32].

**HTDs’** primary role is to develop innovative medicinal products and medical devices and provide requested evidence for regulatory and HTA assessments. However, HTDs clearly stated that their current involvement was inadequate/insufficient and needs to improve. With regards to the development of HTA methodological guidance documents, HTDs have been insufficiently consulted, and their comments, when provided, were largely not taken into account. The current design of product-related scoping processes does not incorporate HTD consultations. Considering their unique insights as main contributors of evidence, opportunities for HTDs to provide input throughout the HTA process, notably during the PICO scoping process and the development of the draft JCA, are called for [33]. Further, the provision of sufficient JSC capacities was raised as an immediate urgency for manufacturers. Having an opportunity to receive guidance on PICO and related evidence requirements could allow them to generate a data package that satisfies both regulatory and HTA needs.

The role of **national HTA bodies** is in transition. While the HTA appraisal and decisions on pricing and reimbursement will remain in the national remit, national HTA bodies will be tasked with both (i) participating in EU HTA assessments, including the preparation of assessment reports and (ii) considering EU assessments within their national environments. To facilitate these tasks, a call for increased capacities for developing the assessment reports and clarification of related financial and political support was defined. Also, details of legislative actions on a national level need to be determined, i.e., which countries require additional national legislative action to adopt the EU HTAR, which have already adjusted their local systems, and which countries have systems in place that allow for adoption without legislative changes.

Various limitations need to be considered with this research. While we aimed to include a sufficient number of representatives of different stakeholders and collaborators in each working group to allow for a lively and balanced discussion, participation was limited to a maximum of 15 people per group and was not equally distributed across groups. As mentioned above, HTA bodies and payers were grouped together in this work due to their common characteristics and challenges based on their role as value assessors and decision-makers in the process, despite the differing remits of their roles’ scope. However, there are likely differing aspects in their viewpoints, which might not be adequately balanced between the break-out groups. In particular, when analyzing the outcomes of discussions (e.g., during a convention), it cannot be excluded that there is bias due to the options of stakeholder availability for participation, participation mode, etc., despite all efforts to balance the representation of stakeholder type, national background, and expertise. Shared information may be incomplete or missing some context, leading to varying interpretations among individual participants. Therefore, this research is of an exploratory nature and serves as a starting point for the “inclusive civil society dialogue” rather than providing definitive insights. Further, in-depth consideration of the sociological and political aspects of this inclusive societal dialogue would be beneficial for optimal stakeholder involvement to achieve the aim of the EU HTAR to improve patient access to lifesaving innovative health technologies.

## 5. Conclusions

During the 2022 Fall Convention of the EAA, a call to action, including the most important activities per stakeholder/collaborator group involved in the EU HTA process, was developed. Addressing an “*inclusive civil society dialogue”*, as suggested by the EC Pharmaceutical Strategy, starts with the implementation of strong and balanced patient involvement in the EU HTA process. Furthermore, medical societies, being the key connecting element in the market access process, should be provided with sufficient capacity to align and standardize European guidelines to allow for comprehensive input in EU HTA civil society dialogue. Efficient and effective collaboration with regulatory authorities throughout the process will be crucial but requires that capacity and technical challenges be addressed. As HTDs are the developers of and experts in the technology to be assessed, options for dialogue and input from HTDs, as well as transparency from horizon scanning via the scoping and assessment phases, are needed. Lastly, the centralized EU HTA procedure can only work if national HTA bodies are willing, have the capacities and experts to participate in assessing health technologies, and the national systems are ready to adopt the centralized reports as part of their procedures. The call to action that we identified is targeted at each of the involved stakeholder/collaborator groups as well as at the Coordination Group, which has the responsibility of overseeing the joint work within the scope of the EU HTAR.

## Figures and Tables

**Table 1:** Role definition of stakeholders and collaborators based on pre-convention questionnaire and working group outcomes.

Stakeholder/Collaborator	Role
Patients	Patients are independent “equal level” stakeholders that should be involved throughout the EU HTA process (i.e., not only in the content, such as specific input on a specific product), including governance, decision-making, dissemination, and advocacy. Involvement both on an EU and national level is important.
Clinicians	Clinicians are to be engaged as advisors adding expertise to the process by: Giving clinical context Defining the standard of care Determining appropriate outcome measurements/endpoints The established and well-functioning cooperative model of the EMA and clinicians should be considered by the EU HTA.
Regulators	Regulatory and HTA bodies have different remits. Regulators collaborate with HTA bodies but are not stakeholders in the meaning of the EU HTA Regulation. Collaboration includes: Contribution throughout the implementation to ensure relevant links through the processes sharing experience, best practices engage in development of capacity and guidance of mutual interest Working together for mutual benefit with the HTA Coordination Group evidence planning/scientific knowledge sharing relevant data and information
HTDs	HTDs develop innovative medicines and provide evidence that allows HTA agencies to conduct a solid and unbiased assessment of the value of the innovation. While communication with HTA agencies to ensure the appropriateness of the submitted evidence is required, this is limited to ensure the independence of HTAs is maintained. Both on a generic methodological level (e.g., input into methodological guidelines) and an applied product-specific level, HTDs contribute scientific excellence throughout the process. HTDs support embedding the EU assessment reports into national appraisals and are addressing any remaining uncertainty.
National HTA bodies and payers	National Expertise Centers of Excellence have a dual role, both as drivers and producers of reports and as users of the EU HTA assessments. National Centers participate in designing and setting up the EU HTA organization and governance structure. They further participate in developing the operational guidelines and they review their internal organization and processes in order to avoid duplication of tasks already performed at the EU level.

***National HTA bodies and payers*** are both drivers and users of the EU HTA assessment and the generated assessment report. Key challenges on their side include low capacity to support the elaboration of EU HTA reports by assessors from national HTA bodies and missing legal frameworks and procedural rules for the adoption of the EU HTA within the national context. Furthermore, addressing heterogeneity across national systems and achieving inclusiveness in all EU countries is seen as a major challenge. EMA: European Medicines Agency; EU HTA: European Union Health Technology Assessment; HTD: health technology developer.

**Table 2:** The most important activities to ensure adequate involvement of stakeholders and collaborators.

Stakeholder/Collaborator	Identified Activities
**Patients**	Training/capacity building and related funding for patient representatives Establish a patient involvement committee (may be part of the EU HTA Stakeholder Network) to define rules, procedures, templates, and methodology for patient input Establish a dedicated “patient unit” at the EC secretariat level
**Clinicians**	Alignment between clinical societies on evidence assessment for a consolidated position to achieve: Narrowing the gaps in methodologies, e.g., pertaining to required methods assessing rare diseases/small populations Management of uncertainty, e.g., context-dependent alignment on acceptable levels of uncertainty More dynamic adoption of new frameworks and methodologies, e.g., basket trials based on the clinical context Capacity building by medical societies: Create a pool of clinicians/societies for involvement in HTA on the EU level Societies to choose their fields and abilities Involve remuneration for consulting Standardization of processes for involvement of clinicians in HTA: Formalized integration of consultation, dialogue, and feedback processes Education on methodologies
**Regulators**	Develop joint guidance documents with HTA: Product specific Therapeutic area specific Methods Align processes and interfaces with HTA bodies under the regulation: Exchange of information in the context of JCA Considering sharing the right data at the right time Respecting remits Expert engagement Shared discussion with HTA bodies on PLEG: Condition-specific evidence generation (registries) Identification of requirements (product specific) Guidance to be developed to engage with other stakeholders Ensure sufficient regulatory capacities for: Early scientific consultation (PICO alignment)
**HTDs**	Rapid introduction of scientific advice within EU HTA at scale—resources, expertise, and agility to respond to the needs Review the scoping process within EU HTA to optimize input and insights from the different stakeholders, especially HTDs: Allow HTDs to propose elements of the PICOs Increase the utility of the process/reports to national HTA agencies Risk with status quo: PICOs not being fit for purpose Develop the scope of the fact-checking process further so that it increases the scientific credibility of the EU report, stakeholder trust, and utilization by national HTA agencies
**National HTA bodies and payers**	Develop joint guidance documents with regulators: Product specific Therapeutic area specific Methods Align processes and interfaces with regulators under the regulation: Exchange of information in the context of JCA Considering sharing the right data at the right time Respecting remits Expert engagement Shared discussion with regulators on PLEG: Condition-specific evidence generation (registries) Identification of requirements (product specific) Guidance to be developed to engage with other stakeholders Ensure sufficient national HTA capacities for: Early scientific consultation (PICO alignment)

EC: European Commission; EMA: European Medicines Agency; EU European Union; HTA: health technology assessment; HTD: health technology developer; JCA: joint clinical assessment; PICO: Population/Intervention/Comparator/Outcomes; PLEG: post-launch evidence generation.

## Data Availability

Raw ranking data are available from the corresponding author upon reasonable request.

## References

[1] (2023). Directorate-General for Health and Food Safety. Overview. https://health.ec.europa.eu/health-technology-assessment/overview_en.

[2] World Health Organization Health Technology Assessment. https://www.who.int/health-topics/health-technology-assessment#tab=tab_1..

[3] Gozzo L., Paterson K., Wong O., Megerlin F., Geldmacher J., Popoli P., Jommi C., Fricke F.-U., De Solà-Morales O., Kamae I. (2022). Towards a European harmonization of health technology assessment recommendations executive paper of European regulatory conference focused on the EU commission proposal to harmonize HTA. Front. Drug Saf. Regul..

[4] Drummond M., Tarricone R., Torbica A. (2022). European union regulation of health technology assessment: What is required for it to succeed?. Eur. J. Health Econ..

[5] European Commission Health Technology Assessment: Commission Welcomes the Adoption of New Rules to Improve Access to Innovative Technologies 2021. https://ec.europa.eu/commission/presscorner/detail/en/IP_21_6771.

[6] The European Parliament and the Council of the European Union Regulation (EU) 2021/2282 of the European Parliament and of the Council of 15 December 2021 on Health Technology Assessment and amending Directive 2011/24/EU2021 22.12.2021. https://eur-lex.europa.eu/legal-content/EN/TXT/PDF/?uri=CELEX:32021R2282&from=EN.

[7] Kristensen F.B., Mäkelä M., Neikter S.A., Rehnqvist N., Håheim L.L., Mørland B., Milne R., Nielsen C.M., Busse R., Lee-Robin S.H., Nielsen C.M., Busse R., Lee-Robin S.H. (2009). European network for health technology assessment, EUnetHTA: Planning, development, and implementation of a sustainable European network for health technology assessment. Int. J. Technol. Assess. Health Care.

[8] Nielsen C.P., Lauritsen S.W., Kristensen F.B., Bistrup M.L., Cecchetti A., Turk E. (2009). Involving stakeholders and developing a policy for stakeholder involvement in the European network for Health Technology Assessment, EUnetHTA. Int. J. Technol. Assess. Health Care.

[9] Julian E., Gianfrate F., Sola-Morales O., Mol P., Bergmann J.F., Salmonson T., Ruof J. (2022). How can a joint European health technology assessment provide an ‘additional benefit’ over the current standard of national assessments? Insights generated from a multi-stakeholder survey in hematology/oncology. Health Econ. Rev..

[10] Julian E., Pavlovic M., Sola-Morales O., Gianfrate F., Toumi M., Bucher H.C., Dierks C., Greiner W., Mol P., Bergmann J.-F. (2022). Shaping a research agenda to ensure a successful European health technology assessment: Insights generated during the inaugural convention of the European access academy. Health Econ. Rev..

[11] Cardone A., van Dyck W., Ermisch M., Gianfrate F., Hebborn A., Julian E., Pavlovic M., Peters S., Price R., Ruof J. (2022). Europe’s Evolving HTA Regulation & It’s Relevance for ‘Beating Cancer’.

[12] Bernardini R., Berntgen M., Van De Casteele M., Desmet T., Dierks C., van Dyck W., Giuliani R., Goettsch W., Guardian M., Van Haesendonck L. (2022). Stakeholder Involvement & Europe’s Evolving HTA Framework.

[13] Van Haesendonck L., Ruof J., Desmet T., Van Dyck W., Simoens S., Huys I., Giuliani R., Toumi M., Dierks C., Dierks J. (2023). The role of stakeholder involvement in the evolving EU HTA process: Insights generated through the European Access Academy’s multi-stakeholder pre-convention questionnaire. J. Mark. Access Health Policy.

[14] Role 2023. https://dictionary.cambridge.org/dictionary/english/role.

[15] European Commission Pharmaceutical Strategy for Europe 2020. https://health.ec.europa.eu/system/files/2021-02/pharma-strategy_report_en_0.pdf.

[16] Cavazza M., Jommi C. (2012). Stakeholders involvement by HTA Organisations: Why is so different?. Health Policy.

[17] Wale J.L., Thomas S., Hamerlijnck D., Hollander R. (2021). Patients and public are important stakeholders in health technology assessment but the level of involvement is low—A call to action. Res. Involv. Engagem..

[18] Vokó Z., Cheung K.L., Józwiak-Hagymásy J., Wolfenstetter S.B., Jones T., Muñoz C., Pokhrel S. (2023). Similarities and Differences between Stakeholders’ Opinions on Using HTA Information Across Five European Countries.

[19] Mitchell G.R., Hartelius E.J., McCoy D., McTigue K.M. (2022). Deliberative Stakeholder Engagement in Person-centered Health Research. Social Epistemol..

[20] Oortwijn W., Husereau D., Abelson J., Barasa E., Bayani D., Canuto Santos V., Culyer A., Facey K., Grainger D., Kieslich K. (2022). Designing and Implementing Deliberative Processes for Health Technology Assessment: A Good Practices Report of a Joint HTAi/ISPOR Task Force. Value Health.

[21] Stakeholders and Communication Division (2022). Engagement Framework: EMA and Patients, Consumers and Their Organisations. EMA/649909/2021:[8 p.]. https://www.ema.europa.eu/en/documents/other/engagement-framework-european-medicines-agency-patients-consumers-their-organisations_en.pdf.

[22] Dierks C., Grimalauskas A., Staeck F., van den Bergh W. (2021). Options of patient participation—A view of the legal framework. Patients and Medical Societies: Additional Expertise for AMNOG.

[23] Schulz-Asche K., Staeck F., van den Bergh W. (2021). The role of patients in the AMNOG procedure from the Green’s point of view. Patients and Medical Societies: Additional Expertise for AMNOG.

[24] Danner M., Staeck F., van den Bergh W. (2021). Patients in the early benefit assessment—A field report. Patients and Medical Societies: Additional Expertise for AMNOG.

[25] Schlegl E., Ducournau P., Ruof J. (2017). Different Weights of the Evidence-Based Medicine Triad in Regulatory, Health Technology Assessment, and Clinical Decision Making. Pharm. Med..

[26] ESMO ESMO-Magnitude of Clinical Benefit Scale 2023. https://www.esmo.org/guidelines/esmo-mcbs.

[27] Cherny N.I., Sullivan R., Dafni U., Kerst J.M., Sobrero A., Zielinski C., de Vries E.G.E., Piccart M.J. (2015). A standardised, generic, validated approach to stratify the magnitude of clinical benefit that can be anticipated from anti-cancer therapies: The European Society for Medical Oncology Magnitude of Clinical Benefit Scale (ESMO-MCBS). Ann. Oncol..

[28] Cherny N.I., Dafni U., Bogaerts J., Latino N.J., Pentheroudakis G., Douillard J.Y., Tabernero J., Zielinski C., Piccart M.J., de Vries E.G.E. (2017). ESMO-Magnitude of Clinical Benefit Scale version 1.1. Ann. Oncol..

[29] Kiesewetter B., Dafni U., de Vries E.G.E., Barriuso J., Curigliano G., González-Calle V., Galotti M., Gyawali B., Huntly B.J.P., Jäger U. (2023). ESMO-Magnitude of Clinical Benefit Scale for haematological malignancies (ESMO-MCBS:H) version 1.0. Ann. Oncol..

[30] Wörmann B., Staeck F., van den Bergh W. (2021). Medical societies in the AMNOG procedure—A “non-quantifiable benefit”?. Patients and Medical Societies: Additional Expertise for AMNOG.

[31] European Medicines Agency European Collaboration between Regulators and Health Technology Assessment Bodies Joint Work Plan (2021–2023) between EMA and European HTA Bodies Facilitated through EUnetHTA212022; EMA/188201/2022:[7 p.]. https://www.ema.europa.eu/en/documents/work-programme/european-collaboration-between-regulators-health-technology-assessment-bodies-joint-work-plan-2021_en.pdf.

[32] Lobker W., Broich K. (2019). Harmonised HTA Assessment: Experiences on the Way to Centralised Approval. European Benefit Assessment—Opportunities and Risks.

[33] EFPIA Joint Statement Pharmaceutical Industry Concerns over the Implementation of the EU HTA Regulation 2022. https://www.efpia.eu/news-events/the-efpia-view/statements-press-releases/joint-statement-pharmaceutical-industry-concerns-over-the-implementation-of-the-eu-hta-regulation/.

